# Rifampin: The Cause of Acute Tubular Injury—A Case Report

**DOI:** 10.1155/crin/7336365

**Published:** 2026-04-28

**Authors:** Muhammad Umer Arif, Areeba Farooq, Harjinder Singh, Muhammad Talha Javaid, Mudassar Hussain

**Affiliations:** ^1^ Department of Microbiology, University of Health Sciences, Lahore, Pakistan, uhs.edu.kh; ^2^ Department of Nephrology, Sahiwal Teaching Hospital, Sahiwal, Pakistan; ^3^ Department of Pathology, Shaukat Khanum Memorial Cancer, Hospital & Research Centre, Lahore, Pakistan

## Abstract

Tuberculosis (TB) presents with fever, night sweats, and cough. The antituberculous therapy (ATT) used to treat TB affects various systems, including the renal system and the hepatobiliary system. ATT‐induced acute tubular necrosis (ATN) can have various presentations, such as fever, vomiting, diarrhea, abdominal pain, oliguria, and cola‐colored urine. We present a case of a 56‐year‐old patient using ATT. He presented with the complaint of altered mental status, vomiting, jaundice, and cola‐colored urine. Labs revealed elevated liver enzymes, elevated creatinine, anemia, and thrombocytopenia. The autoimmune profile was normal; however, the biopsy revealed ATN. In addition to supportive management, hemodialysis and steroid therapy were initiated. Discontinuation of rifampin along with continuation of modified ATT therapy led to improved renal function tests, platelet counts, and Hb levels.

## 1. Introduction

Tuberculosis (TB) is the leading health challenge in the developing world. It affects more than a million people per year in Asia. The first‐line management of TB includes isoniazid, rifampin, and pyrazinamide in the intensive phase for 2 months, followed by a continuous phase of 4 months with rifampin, isoniazid, and ethambutol [1]. These drugs cause various side effects. Anti‐TB therapy particularly causes hepatic and renal toxicity. Rifampin has been noted to cause renal toxicity with acute interstitial nephritis, acute tubular necrosis (ATN), rapidly progressive glomerulonephritis, and light chain proteinuria [2]. The deleterious action of isoniazid, which has functional and structural effects on renal tissue, has also been reported [3]. If renal clearance is decreased, ethambutol causes optic neuritis, which is its major side effect [4]. Thus, this case emphasizes early signs of renal failure in patients taking antituberculous therapy (ATT), its management, and prognosis. Even if ATT can adversely affect renal function, a primary physician must be aware of patients currently taking or previously taking these medications and how to proceed with their management.

## 2. Case Presentation

A 56‐year‐old male developed fever, weight loss, and cough for 2.5 months. A chest X‐ray revealed a granuloma. He tested positive for acid‐fast bacillus in his sputum. He was diagnosed with pulmonary TB and started on the anti‐TB drugs isoniazid, rifampin, and pyrazinamide. A detailed medication history was obtained from both the patient and his family. They consistently confirmed that the ATT was taken continuously as prescribed, with no missed doses, interruptions, or unsupervised reinitiation of rifampin prior to presentation.

He was in a usual state of health 2 days back, 1 month after initiating the therapy, when he presented with drowsiness, which was gradual in onset, progressive in nature, and associated with irritability and irrelevant talk noticed by his family members, who brought him to the hospital. He was placed in Grade 2 encephalopathy.

He also had a complaint of constipation for 2 days, which was sudden in onset and progressive in nature. He normally had a daily bowel movement, but for the last 2 days, he had to strain significantly. Although he was able to pass flatus, he was unable to pass stool, which caused him much discomfort. As noticed by his family, the patient also experienced worsening of yellowing of the skin and eyes for the last few days in addition to cola‐colored urine for the last 2 days, which was sudden in onset and progressive.

On general examination, he was found to be pallor, jaundiced, and lethargic. His vital signs at the time of presentation were a pulse of 73 bpm, a BP of 110/70, a temperature of 38.0 degrees Celsius, a respiratory rate of 18, and an oxygen saturation of 95% in room air. On systemic examination, the GCS was 14/15 (E4V4M6), Grade 2 encephalopathy, and basal crepts were observed on both sides of the lungs via auscultations, and tenderness was detected by palpating the abdomen. The rest of the examination was unremarkable.

Urgent labs were ordered, which included complete blood count (CBC), liver function test (LFT), renal function test (RFT), blood sugar random (BSR) test, serum electrolytes, clotting profile, blood group C cross match, and hepatitis screening. His laboratory investigations are given in Table 1.

**TABLE 1:** Laboratory investigations at the time of admission.

Lab values	Results	Reference range
White blood cell count	30 × 10^9^ µ/L	4.5–11.0 × 10^9^ µ/L
Hemoglobin	6.5 g/dL	13.5–17.5 g/dL
Platelet count	66,000 µL	150,000–400,000 µL
Bilirubin	31.7 mg/dL	0.1–1.0 mg/dL
Alanine transaminase	1970 U/L	8–20 U/L
Aspartate transaminase	786 U/L	8–20 U/L
Alkaline phosphatase	179 U/L	44–147 U/L
Creatinine	12.4 mg/dL	0.3–1.1 mg/dL
Urea	234 mg/dL	15–40 mg/dL
Total protein	5.8 g/dL	6.0–7.8 g/dL
Albumin	3.0 g/dL	3.5–5.5 g/dL
Prothrombin time	15 s	11–15 s
Active partial thromboplastin time	37 s	25–40 s
International normalized ratio	1.11	0.8–1.1
Sodium	145 mmol/L	135–145 mmol/L
Potassium	5.2 mmol/L	3.5–5.0 mmol/L
Chloride	107 mmol/L	95–105 mmol/L
Lactate dehydrogenase	123 U/L	140–280 U/L
Hepatitis B surface antigen	Negative	
Anti‐hepatitis C virus	Negative	
Anti‐human immunodeficiency virus	Negative	
Blood sugar random	177 mg/dL	Normal: < 140 mg/dL; prediabetes: 140–199 mg/dL; diabetes: ≥ 200 mg/dL

Furthermore, his urine analysis and immunologic–serologic workup results are given in Tables 2 and 3, respectively.

**TABLE 2:** Urine analysis results.

Urine analysis	Results	Reference range
Protein	+++	Negative to trace
White blood cell/pus cells	3‐4/hpf	0–5/hpf
Red blood cells	20–32/hpf	0–2/hpf
Blood	3+	Negative

**TABLE 3:** Immunologic and serologic workup results.

Lab values	Results	Reference range
Anti‐neutrophilic antibodies	Negative	Negative: < 1:40 titer or < 1.0 index; positive: ≥ 1:40 titer or ≥ 1.0 index
Cytoplasmic anti‐neutrophil cytoplasmic antibodies	Negative	Negative: < 20 IU/mL or no fluorescent pattern (IIF); positive: ≥ 20 IU/mL or positive fluorescence
Perinuclear anti‐neutrophil cytoplasmic antibodies	Negative	Negative: < 20 IU/mL or no fluorescent pattern (IIF); positive: ≥ 20 IU/mL or positive fluorescence
Complement component 3	130 mg/dL	90–180 mg/dL
Complement component 4	28 mg/dL	10–40 mg/dL
Anti‐streptolysin O titer	Negative	Negative: < 200 IU/mL; positive: ≥ 200 IU/mL

**FIGURE 1:**
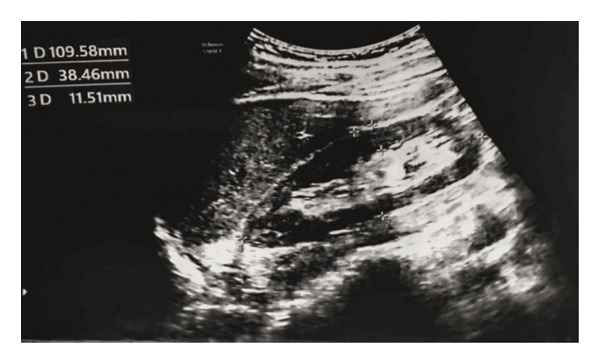
Renal ultrasound showing preserved corticomedullary differentiation.

Foley catheters were passed to monitor the patient’s urine output, which was 350 mL/12 h. According to the RIFLE criteria, he was classified into an “injury” class. On the basis of his physical examination findings, creatinine and urine output, a hemodialysis session was started on the 2nd day of admission, and 4 sessions of hemodialysis were subsequently conducted over the next 10 days to improve his creatinine levels, urine output, and overload state. Ultrasound revealed bilateral increased renal echogenicity and preserved corticomedullary differentiation, sludge in the gallbladder, floating echogenic foci in the portal vein, and mild free fluid in the pelvis. The abovementioned ultrasound findings are shown in Figure 1.

After the initial baseline and first session of hemodialysis, given his markedly increased white blood cell (WBC) count of 30, he was given additional treatment. This included broad‐spectrum antibiotic therapy with Tanzo (tazobactam/piperacillin) 2.25 g, hepatobiliary support with Hepamerz injection and Urso 500 mg capsule, proton‐pump inhibitor therapy with Vify (omeprazole) 40 mg injection, Duphalac syrup and Kleen Enema for constipation, intravenous fluids, and one unit of packed red blood cells. In view of his acute kidney injury, Deltacortil 5 mg was also added as corticosteroid therapy..

The patient’s laboratory tests were performed on a daily basis to monitor his condition, as shown in Table 4. Although the patient’s clinical condition and laboratory parameters improved, several values remained abnormal. In particular, the D‐dimer level remained elevated at 4500 U/L.

**TABLE 4:** Laboratory investigations over the course of several days.

Lab values	Baseline	1st day	3rd day	5th day	7th day	9th day	11th day
Creatinine	0.9	12.4	9.1	—	6.4	6.3	5.8
Urea	—	234	301	—	220	181	215
Bilirubin (total mg/dL)	—	31.7	—	—	—	39.7	29.1
Aspartate transaminase (U/L)	—	786	—	270	—	86	70
Alanine transaminase (U/L)	—	1970	—	1265	—	799	575
Sodium (mmol/L)	—	145	145	135	135	141	143
Potassium (mmol/L)	—	5.2	5.2	5.6	4.9	5.3	4.4
Chloride (mmol/L)	—	107	107	103	106	106	107
White blood cell count (× 10^3^/μL)	—	30	—	20.1	12.5	6	—
Platelet count (× 10^3^/μL)	254	66	—	34	37	35	—
Hemoglobin (g/dL)	12	6.5	5.6	6.8	7.3	10.2	—

Despite the presence of cola‐colored urine observed clinically, a renal biopsy revealed that no proliferative glomerulonephritis or crescentic glomerulonephritis was present. The histopathological findings are provided in Supporting Information 1. The tubules showed mild atrophy characterized by dilatation, loss of brush borders, hyperchromasia of nuclei, loss of nuclei, sloughing of epithelial cells, and flattening of the epithelium. Renal biopsy demonstrated moderate acute and chronic interstitial nephritis with moderate acute tubular injury. There was a prominent interstitial inflammatory infiltrate predominantly composed of neutrophils, rare eosinophils, and lymphocytes. Tubular changes included marked dilatation, loss of brush borders, epithelial flattening, and luminal sloughing. There was no evidence of pigment nephropathy, hemoglobin or red blood cell casts, or thrombotic microangiopathy. Vessels were unremarkable, and glomerular basement membranes displayed no double contouring. These histopathological findings are consistent with drug‐induced interstitial nephritis and tubular injury, associated with rifampin exposure being a well‐recognized trigger. The detailed diagnostic report is available in Supporting Information 2.

## 3. Discussion

The management of TB in kidney patients has not been clearly outlined, and no trials have provided enough evidence to guide treatment. Anti‐TB drugs are generally well tolerated in CKD patients, but drug‐related adverse effects are also frequent and secondary to reduced renal elimination [5]. Furthermore, managing AKI in the setting of anti‐TB therapy is challenging, and there are no clear recommendations. With the case mentioned, we intend to highlight the unfavorable effects of ATT that may pose a threat to life and require a high index of suspicion and timely intervention to prevent any unfortunate incident.

The primary drugs used in the anti‐TB therapy, rifampin, isoniazid, ethambutol, and pyrazinamide, are all associated with AKI [1, 6, 7]. Pathological abnormalities caused by rifampin nephrotoxicity include acute interstitial nephritis, rapidly progressive glomerulonephritis, ATN, and light chain proteinuria [2]. Rifampin instigates a hypersensitivity reaction, resulting in the formation of anti‐rifampin antibodies, which form immune complexes, and these complexes are deposited in vessels and the interstitium, causing ischemic ATN and AIN, respectively [7]. There are also a few causes of RPGN caused by rifampin, which responds to methylprednisolone [8].

Owing to the reduced activity of the kidney, other ATT drugs also cause catastrophic effects. The toxicity of isoniazid can lead to reduced production of gamma‐aminobutyric acid (GABA), which is an inhibitory neurotransmitter and may manifest as seizures [9]. Decreased removal of pyrazinamide can cause hyperuricemia. Pyrazinoic acid, the active metabolite, competes with uric acid for renal clearance and results in the exacerbation of gout in susceptible individuals. Ethambutol is notorious for causing optic neuritis because of its toxicity. In addition, it may also lead to hyperuricemia, peripheral neuropathy, and AIN [4]. Renal insufficiency, tubular damage, diffuse interstitial fibrosis, sclerosis glomerular damage, and tubular atrophy have also been reported in some cases [1]. The most crucial management step in ATT related to AKI is the immediate discontinuation of the culprit drug [7]; steroids can be used in severe cases; however, their benefit has not yet been proven.

As we discuss this case and review the literature, the patient used isoniazid, rifampin, and pyrazinamide. When the patient initially started taking the medications, he had normal Hb and platelet levels but increased WBC levels. One month after starting these drugs, the patient developed anemia and thrombocytopenia. Moreover, he developed ATN and drug‐induced hepatitis. While the LDH levels were within the normal range, D‐dimer levels increased, which is an indicator of acute kidney dysfunction [10].

Anemia may be the consequence of chronic infection, as the progression of the pulmonary TB leads to anemia [11]. This may be due to iron deficiency or chronic inflammation. Treating anemia with iron therapy during the active phase of pulmonary TB is not recommended [12]. Treating the underlying cause by continuation of ATT results in settling anemia in such patients. However, in this case, due to severe anemia (Hb < 7.9 g/dL), pack cells were transfused.

The patient developed thrombocytopenia, which may be the later consequence of ATT. Rifampicin‐dependent antibodies interact with the I‐antigen on the RBC surface and glycoprotein IX on platelets, leading to complement‐mediated destruction and causing intravascular hemolysis and thrombocytopenia, respectively. Owing to severely declining platelet counts, FFPs were transfused into the patient. Modified ATT therapy was also used in patients in whom rifampin was excluded, and follow‐up revealed an improved platelet count. The patient underwent treatment consisting of an antibiotic combination of piperacillin + tazobactam intravenously (IV), a proton‐pump inhibitor IV, and fluid dextrose‐saline with Hepamerz IV. Along with hemodialysis, steroids were initiated in this patient. Steroids hasten renal recovery; however, they should be used in severe cases, as their benefits have not yet been proven, and they may exacerbate the underlying TB infection due to immunosuppression [12]. Although hemolysis and thrombocytopenia were considered, renal biopsy revealed no evidence of pigment nephropathy, hemoglobin casts, or thrombotic microangiopathy, making these mechanisms unlikely contributors to AKI (see Supporting Information 1).

## 4. Conclusion

Eight weeks after the cessation of the rifampin and following hemodialysis sessions, renal function improved, and the creatinine level decreased to the baseline level. LFTs had also normalized, resulting in sustained clinical and laboratory improvement. Once organ function stabilizes, ATT is typically reintroduced sequentially at full doses, with agents added every 2‐3 days to allow close laboratory monitoring, generally beginning with ethambutol, followed by rifampin, and then isoniazid. If rifampin cannot be safely reintroduced, continuation therapy should be extended accordingly [13]. In our case, rifampin was not reintroduced due to the severity of renal and systemic adverse effects, and a modified ATT regimen was pursued.

Similarly, 8 weeks after the abovementioned treatment and cessation of rifampin, the patient was followed up, and his laboratory results revealed improved Hb levels, without further active management for anemia. The patient’s platelet level also improved secondary to the discontinuation of rifampin.

Thus, this case highlights that ATT‐induced ATN can even occur with continuous dosing, first exposure, and no prior history of ATT use. The key learning point is the immediate action of medical practitioners and vigilant monitoring of the renal profile of such patients because ATT‐induced ATN poses a health risk to the patient and may further lead to discontinuation of treatment, resulting in resistance to drugs. In addition, our case suggests that many immune‐mediated mechanisms and pathophysiological processes are involved in ATT‐induced AKI, and further investigations are warranted to understand and improve our strategies. Early discontinuation of rifampin from ATT led to improvements in RFTs, platelet counts, and Hb levels without targeted management for anemia and thrombocytopenia. Clinicians must suspect drug‐induced renal toxicity in patients using ATT because early diagnosis and intervention can lead to improved prognosis.

Given the temporal relationship, biopsy findings (see Supporting Information 1), and exclusion of alternative mechanisms, rifampin‐induced acute interstitial nephritis with tubular injury was considered the most likely etiology.

## Funding

No funding was received for this manuscript.

## Consent

No written consent has been obtained from the patient as there are no identifiable patient data included in the case report.

## Conflicts of Interest

The authors declare no conflicts of interest.

## Supporting Information

Additional supporting information can be found online in the Supporting Information section.

## Supporting information


**Supporting Information 1** Histopathological biopsy findings (Supporting Information—Biopsy.pdf).

**Supporting Information 2** Detailed diagnostic report (Supporting Information—Report.pdf).

## Data Availability

The data that support the findings of this study are available on request from the corresponding author. The data are not publicly available due to privacy or ethical restrictions.
