# Supplementary material for: Rifampin: The Cause of Acute Tubular Injury—A Case Report
Source: Case Rep Nephrol. 2026 Apr 28;2026:7336365. doi: 10.1155/crin/7336365 (PMC13125855; doi:10.1155/crin/7336365)
Supplement: Supplementary file 1 — Supporting Information 1 Histopathological biopsy findings (Supporting Information—Biopsy.pdf). [file CRIN-2026-7336365-s001.pdf]

Specimen Nature: RENAL BIOPSIES

Specimen Site: KIDNEY

History: Known case of pulmonary TB and patient is on ATT. Diagnosis; AKI/ATN/RPGN/AIN. Labs; Hb 9.4-6.9-6.4-7.3, TLC 13.8, platelet 381, urea 154 raised, creatinine 0.9 o 1.1 to 12.4 to 10.3 to 11.4 to 12.5 raised, sodium 132, postpartum 5.4, C3, C4, ANA, ANCA, CP normal, urinary examination protein positive, RBC 20-32, pus cells 3-4, blood +++, sugar positive, minor markers negative.

Gross: SPECIMEN #1 (KIDNEY, RENAL BIOPSY):
Specimen container is labeled with the patient's name and medical record number and is unmarked for site. Received in formalin is a single intact core measures 7 mm. The entire specimen is submitted in single block.

SPECIMEN #2 (KIDNEY, RENAL BIOPSY):
Specimen container is labeled with the patient's name and medical record number and is unmarked for site. Received in normal saline is a single core and a core fragment measuring 7 mm and 2 mm respectively. The entire specimen is submitted in Michelle solution.

Micro: Renal Biopsy Reporting Checklist

Glomeruli: (Evaluated on Hematoxalin & Eosin, JMS, PAS stains)
Number of Glomeruli: 6
Glomerular Change: The biopsy is composed of single core of renal parenchyma with cortex only. One glomerulus is globally sclerotic. The rest of all glomeruli are predominantly unremarkable at the level of light microscopy. There are no proliferative changes comprising neutrophils, endocapillary proliferation or extracapillary proliferation. The glomerular basement membranes are unremarkable. No spikes or double contouring is seen. No segmental sclerosis is present and the Bowman's capsule entirely unremarkable.

INTERSTITIUM (EVALUATED ON H&E AND TRICHROME):
Inflammation: Moderate acute and chronic inflammation comprising significant neutrophils with rare eosinophils and lymphocytes are seen.
Fibrosis: Mild interstitial fibrosis 15-20% is present.

TUBULES:
Mild tubular atrophy 15-20% is seen. In addition, there are significant features of acute tubular injury characterized by dilatation, loss of brush border, hyperchromasia of nuclei, loss of nuclei, sloughing off of epithelial cells and flattening/simplification of epithelium.

VESSELS:
Unremarkable.

IMMUNOFLUORESCENCE:
IgG: Negative
IgA: Negative
IgM: Negative
C1q: Negative
C3: Negative
Kappa: Negative
Lambda: Negative

Diagnosis: KIDNEY, RENAL BIOPSY:
Moderate acute and chronic interstitial nephritis, see note.
Moderate acute tubular injury.
Mild interstitial fibrosis and tubular atrophy 15-20% of cortex.
No proliferative glomerulonephritis is identified.

Note: No proliferative glomerulonephritis or crescentic glomerulonephritis is seen. The overall features are consistent acute/chronic interstitial nephritis with acute tubular injury.Drugs or hypovolemia may be the case of this condition. Please correlate with clinical findings.

For any query regarding diagnosis, treating physician can contact at 03000453078 between 8:00 AM to 5:00 PM (working days only).

SNOMED: T-71000 M-68130
