# Supplementary material for: Rifampin: The Cause of Acute Tubular Injury—A Case Report
Source: Case Rep Nephrol. 2026 Apr 28;2026:7336365. doi: 10.1155/crin/7336365 (PMC13125855; doi:10.1155/crin/7336365)
Supplement: Supplementary file 2 — Supporting Information 2 Detailed diagnostic report (Supporting Information—Report.pdf). [file CRIN-2026-7336365-s002.pdf]

BIOPSY REPORT
Specimen Nature: RENAL BIOPSIES
Specimen Site: KIDNEY

History: Known case of pulmonary TB and patient is on ATT. Diagnosis: AKI/ATN/RPGN/AIN. Labs: Hb 9.4-6.9-6.4-7.3, TLC 13.8, Platelet 381, Urea 154 (raised), Creatinine 0.9 to 1.1 to 12.4 to 10.3 to 11.4 to 12.5 (raised), Sodium 132, postpartum 5.4, C3, C4, ANA, ANCA CP normal, Urinary examination protein positive, RBC 20-32, pus cells 3-4, blood +++, sugar positive, minor markers negative.

Gross: Specimen 1: Received in formalin; a single intact core measuring 7 mm. The entire specimen submitted in single block.
Specimen 2: Received in normal saline; a single core (7 mm) and a core fragment (2 mm) submitted in Michel's solution.

Microscopic Evaluation:

Glomeruli: 6 glomeruli evaluated. One is globally sclerotic; the remainder are predominantly unremarkable under light microscopy. No proliferative changes comprising neutrophils, endocapillary proliferation or extracapillary proliferation noted. Basement membranes are unremarkable; no spikes or double contouring seen. No segmental sclerosis is present and the Bowman's capsule entirely unremarkable.

Interstitium: Moderate acute and chronic inflammation with significant neutrophils, rare eosinophils and lymphocytes. Mild interstitial fibrosis (15-20%) is present.

Tubules: Mild tubular atrophy (15-20%). Significant features of acute tubular injury (ATI) characterized by dilatation, loss of brush border, hyperchromasia of nuclei, sloughing off of epithelial cells, and flattening/simplification of epithelium.

Vessels: Unremarkable.

Immunofluorescence (IF):

IgG: Negative

IgA: Negative

IgM: Negative

C1q: Negative

C3: Negative

Kappa: Negative

Lambda: Negative

Final Diagnosis

1. Moderate acute and chronic interstitial nephritis.
2. Moderate acute tubular injury.
3. Mild interstitial fibrosis and tubular atrophy (15-20% of cortex).

Note: No proliferative glomerulonephritis or crescentic glomerulonephritis is seen. The overall features are consistent acute/chronic interstitial nephritis with acute tubular injury. Drugs or hypovolemia maybe the case of this condition.
